# Effects of Heat Stress on Construction Labor Productivity in Hong Kong: A Case Study of Rebar Workers

**DOI:** 10.3390/ijerph14091055

**Published:** 2017-09-12

**Authors:** Wen Yi, Albert P. C. Chan

**Affiliations:** 1 School of Engineering and Advanced Technology, College of Sciences, Massey University, Auckland 0745, New Zealand; yiwen96@163.com; 2 Department of Building and Real Estate, The Hong Kong Polytechnic University, Hung Hom, Kowloon, Hong Kong, China

**Keywords:** heat stress, construction labor productivity, steel bar fixing

## Abstract

Global warming is bringing more frequent and severe heat waves, and the result will be serious for vulnerable populations such as construction workers. Excessive heat stress has profound effects on physiological responses, which cause occupational injuries, fatalities and low productivity. Construction workers are particularly affected by heat stress, because of the body heat production caused by physically demanding tasks, and hot and humid working conditions. Field studies were conducted between August and September 2016 at two construction training grounds in Hong Kong. Onsite wet-bulb globe temperature (WBGT), workers’ heart rate (HR), and labor productivity were measured and monitored. Based on the 378 data sets of synchronized environmental, physiological, construction labor productivity (CLP), and personal variables, a CLP-heat stress model was established. It was found that WBGT, percentage of maximum HR, age, work duration, and alcohol drinking habits were determining factors for predicting the CLP (adjusted *R*^2^ = 0.68, *p* < 0.05). The model revealed that heat stress reduces CLP, with the percentage of direct work time decreasing by 0.33% when the WBGT increased by 1 °C. The findings in this study extend the existing practice notes by providing scientific data that may be of benefit to the industry in producing solid guidelines for working in hot weather.

## 1. Introduction

The global average surface temperature increased by 0.74 °C ± 0.18 °C between 1906 and 2005, which is largely than that of 0.6 °C ± 0.2 °C between 1901 and 2000 due to the additional warming years [1]. According to the global climate report 2011–2015 (World Meteorological Organization), the average global temperature increased by up to 1 °C in 2015. Extreme high temperatures and heat waves frequently appear year-round at low latitudes, and in summer time at middle latitudes [2]. There have been many high-impact events due to global warming, including the 2010–2012 East African drought, resulting in more than 258,000 weather-related fatalities; and the 2015 heat waves in Pakistan and India, which led to approximately 4100 mortalities [3]. The climate is getting warmer, and prolonged periods of excessive heat have spread to large parts of Australia, Asia, and Europe [4]. People with cardiovascular and heart-disease are more vulnerable, and would be under higher risk of heat-related illness due to global warming [5,6].

Excessive heat exposure is a well-known occupational health hazard that causes illnesses ranging from cramps to death. Exposure to intense or prolonged heat and humidity can reduce workers’ enthusiasm and concentration for their work, increase their irritability, and lead to heat-related illnesses [7,8]. In the United States, there were at least 30 workers who died annually from heat-related illnesses and injuries between 2003 and 2012 [9]. Data from the Center for Construction Research and Training of the US shows that 17 construction workers died in 2015 as a result of heat-related conditions [10]. Construction workers, particularly those who undertake scaffolding tasks, steel bar fixing, structural steel erection, formwork, concrete pouring, are considered the most vulnerable population to heat stress. This is understandable, because they usually have to work in open areas without shading when performing their daily operations. In fact, the temperature of a construction site can be much higher than the air temperature; Chan et al. reported the temperature of a construction site could be as high as 45 °C when the air temperature was 32 °C [11]. To protect construction workers from heat stress, prevention measures including work-break cycles, work arrangement, and cool down facilities providing drinking water or sports drinks were suggested to safeguard the workers’ health and well-being in hot environments [12,13].

Excessive occupational heat stress not only affects the individual, but also influences work productivity and performance, and even the local economy [14,15]. Dunne et al. estimated that environmental heat stress has already reduced the global labor capacity significantly in peak months with a further predicted reduction of 80% by 2050 [16]. According to the assessment of productivity loss based on heat exposure physiological models, Zander et al. estimated that productivity might reduce by 11–27% at the end of this century in hot areas (e.g., the Caribbean and Asia) [17]. Altinsoy and Yildirim found labor productivity in agriculture and construction is expected to diminish seriously in western Turkey, which may reach up to 52% labor productivity loss during the summer between 2071 and 2100 [18].

Productivity is a significant component in the construction industry around the world. Labor productivity in the construction industry has drawn great attention, as the industry faces multiple problems related to its workforce. Construction labor productivity (CLP) is often influenced by variations in work conditions. The complex relationship between CLP and hot environments has received attention in academia for decades, and yet interest in this field has never diminished. Based on a field study, Grimm and Wagner explored the association between CLP and thermal environment which combines air temperature and relative humidity [19]. This study showed that humidity can be a significant factor at both high and low temperatures, and that workmanship quality could decline at relatively high temperatures. Based on a database with a large number of air temperature and humidity values, a non-linear association between CLP and thermal environments was found [20]. The output of this relationship agrees reasonably well with a CLP model proposed by Thomas and Yiakoumis [21]. Nevertheless, both of these models have been criticized for ignoring the effect of relative air movement. Hancher and Abd-Elkhalek introduced a more sophisticated CLP model that took both work condition and climatic environment into account [7]. This model utilizes a more integrated heat stress index—wet bulb globe temperature (WBGT)—to examine the impacts of hot environments on human perceptional and physiological response. However, this productivity model did not allow for the influence of clothing ensembles on the worker’s thermal sensation. Clothing ensemble plays an important role in transferring the heat between the climatic environment and human body [22]. Earlier studies indicated that a comfortable thermal environment could produce better work performance [18,19,23]. Quantitative analysis methods such as multiple linear regression analysis have been used to develop productivity models.

More recently, Srinavin and Mohamed established a model for estimating productivity for construction tasks in Thailand [23]. The proposed model takes all these thermal parameters, namely, ambient temperature, relative humidity, radiant temperature and air velocity, the nature of construction task as well as clothing of the worker into consideration. Li et al. evaluated the effects of heat stress on CLP in Beijing, China [24]. This model explored the relationship between CLP and WBGT as well as workers’ personal information (i.e., age, body fat, working experience). It was found that CLP reduced by 0.57% when the WBGT elevated by 1 °C. Although interest in assessing the impacts of hot temperatures and CLP has increased, less evidence on the effect of heat stress on CLP is available in Hong Kong. Due to the high-density city and the subtropical climate, Hong Kong’s summer is hot and humid. The objectives of this study are to (1) investigate the heat stress level of construction sites and heat strain of construction workers; (2) develop a model for predicting CLP in hot and humid environments. Since rebar fixing is one of the most labor-intensive and time-consuming activities [25], heat stress can seriously affect rebar fixers’ productivity and threaten their health and safety. This study selected the steel bar fixing as the prototype trade to develop a more sophisticated methodology.

## 2. Material and Methods

### 2.1. Participants

Fourteen local male steel bar fixers participated in the field studies. The sample size was determined based on the significance level (set alpha = 0.05), statistical power (set 1 − beta = 0.8), required treatment difference (difference in core temperature and heart rate, determined by literature review and expert evaluations), and standard deviation. The participants had had no major health problems in the past (e.g., hypertension, diabetes, cardiovascular problem, or neurological disease) or symptoms of heat-related illness (e.g., confusion, drenching sweats, headache, fainting, nausea, shortness of breath, or cramps). All the participants were engaged in steel bar fixing work and had acclimatized to work in a hot environment for more than 1 month (from June to August 2016). All of the participants were provided with a type of construction uniform that consisted of a polo T-shirt and a pair of long trousers. The thermal insulation (It) and evaporative resistance (Ret) parameters of the construction uniform were 0.16 m^2^·K/W and 16.96 Pa·m^2^/W, respectively. Participants were briefed on the purposes and procedures of the field studies, and each provided a signed written consent form. Participants were informed that participation in the study would be voluntary and that they could withdraw at any time.

### 2.2. Measurements

#### 2.2.1. Climatic Heat Stress

**Figure 1:**
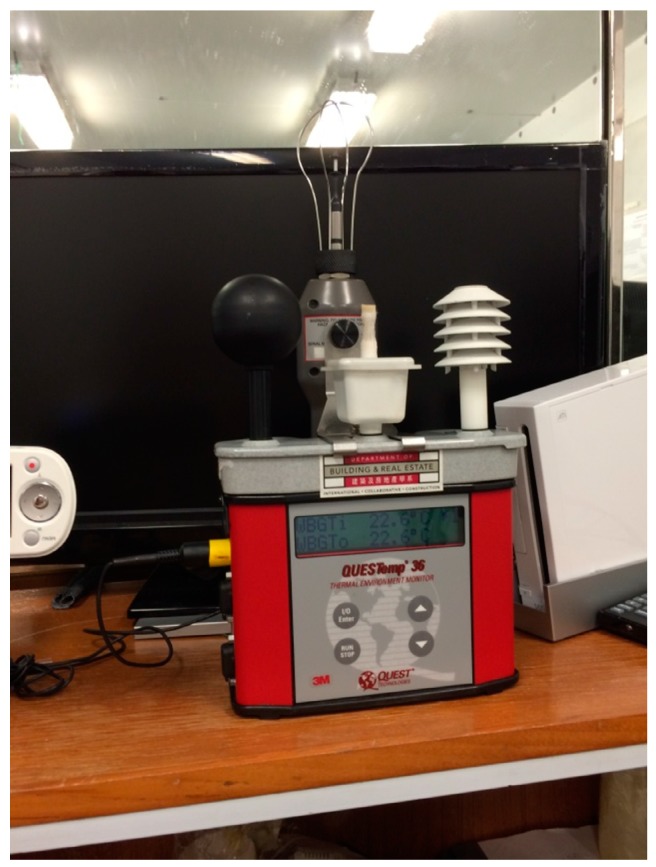
Heat stress monitor (QUESTemp° 36, 3M, North Ryde, Australia).

The WBGT is a kind of apparent temperature that integrates the effects of ambient temperature, relative humidity, wind chill, as well as solar radiation. It is the most widely accepted and used method for measuring environmental variables on occupational heat stress [26]. The WBGT has been recognized by several agencies (e.g., the American Conference of Governmental Industrial Hygienists, National Institute for Occupational Safety and Health, International Organization for Standardization) as a safety index to set limits in industrial workplaces [11]. Furthermore, Yi and Chan revealed that WBGT has the highest validity in forecasting the impacts of occupational heat exposure in the construction industry [27]. In this study, we used an instrument (QuesTemp 36, Quest Technologies, Onoconomac, WI, USA) to measure and monitor the WBGT (Figure 1). The heat stress monitor is capable of measuring four environmental variables (ambient or dry bulb temperature, globe temperature, relative humidity, and natural wet bulb temperature) at 1 min intervals. The WBGT can be simultaneously computed and indicated by these four parameters.

#### 2.2.2. Heart Rate

**Figure 2:**
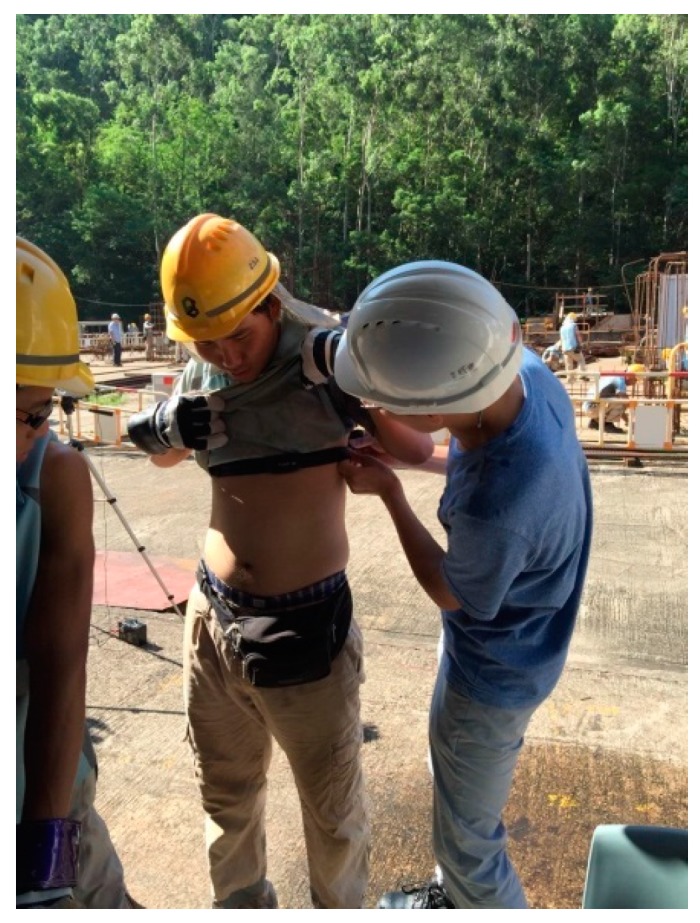
Heart rate belt (Polar Wearlink^®^, Polar Electro Oy, Kempele, Finland).

The combination of environmental heat stress, metabolic rate, and clothing ensembles can lead to an increase in body temperature. An increase of body temperature prompts heat transfer from the human body to the environment in order to maintain a stable body temperature [28]. Heart rate (HR), sweating rate, body core temperature, and skin temperature have been recognized as the main indicators for heat strain. Heat strain is related to the age, weight, physical fitness, medical condition, and degree of acclimatization. As the earliest response of physiological strain [29], HR has been widely used to evaluate the physiological strain of industrial workers exposed to heat stress [30]. In this study, we used a portable heart rate belt (Polar WearLink + chest belt, Polar Electro Oy, Kempele, Finland) to measure the HR of construction workers (Figure 2). This device can continuously measure the real-time HR for every 5 s interval. The hourly HR value was used to identify the physiological strain level. The maximum heart rate (HR_max_) is the highest heart rate a person can safely achieve through exercise stress. We estimated the HRmax by using the age-predicted equation [31]:

HR_max_ = 220 − Age (1)

#### 2.2.3. Construction Labor Productivity

Construction labor productivity is widely considered to be the ratio of a volume measure of output to a volume measure of input. Maloney asserted that the utilization of craft work time can be used to reflect the ‘organizational imposed constraints’ that impede the enhancement of productivity [32]. The best way to improve labor productivity is to examine the work process from the worker’s perspective. Many studies have suggested that the percentage of time spent in productive activities can be considered an effective indicator to assess the CLP [33]. In this study, we measured the labor productivity by adopting the method proposed by the AACE International. AACE International classified work activities into three categories: (1) direct task—assigned work that needs specific efforts or the use of tools/equipment that productively and directly contribute to the completion of the task scope; (2) indirect task—support work or assistance that are not directly conducive to the completion of the task scope; and (3) non-productive time—personal time and non-utilization time due to work stoppage from any cause. Based on the AACE International method, we carried out continuous direct observations throughout the work-day of the participating steel bar fixers. Further modifications were made by the onsite supervisors and foremen. Table 1 shows the breakdown of direct, indirect, and non-productive activities for rebar workers.

### 2.3. Experimental Procedure

Field studies were conducted between August and September 2016 at two construction training grounds in Hong Kong. Construction work included daily morning (8:00–12:00) and afternoon sessions (13:00–16:30), with a one-hour break at noon (12:00–13:00). The heat stress monitor was set in the workplace to measure the heat stress level at the construction site. Fourteen (14) rebar workers were recruited from seven steel bar-fixing groups. They were asked to get together at 8:00 to put on a heart rate belt. Then they rested in a shaded space for 30 min to stabilize their heart rate. During this rest period, participants were asked to report their demographic information, including gender, age, and personal health data, including smoking habits, alcohol drinking habits, and sleeping hours. The body mass, height, BMI, resting heart rate, and tympanic temperature of the participants were measured. All the participants were Chinese males, with an average age of 29 ± 3.32. The body mass, height, BMI, resting heart rate, and tympanic temperature of the participants were 62 kg ± 6.75 kg, 171 cm ± 5.63 cm, 21.2 ± 2.17, 72 ± 5 beats/min, and 36.16 °C ± 0.63 °C, respectively. Of all 14 participants, 50% did not smoke at all, 21% had 1 to 4 cigarettes per day, and 29% had 5 cigarettes or more per day; 43% did not consume alcoholic drinks at all, 50% consumed one cup of red wine/white spirit/beer per day, and 7% consumed two cups or more red wine/white spirit/beer per day. The participants slept 7.2 h ± 0.7 h per day.

During the experiment, workers fixed steel bar reinforcements as their normal work routine. During the rest periods, rebar workers were permitted to take water and wipe off sweat as usual. The real-time HRs of the participants were displayed on the computer. The participants were asked to stop when their HR reached 95% of the age-predicted HR_max_, or when they self-reported reaching exhaustion (whichever came first). Rest periods of 15 and 30 min were arranged between bouts of work in the morning work session and afternoon work session (i.e., 10:00–10:15 in the morning and 15:00–15:30 in the afternoon) [27]. Thus, six test bouts were included, i.e., 1st morning work (MW_1_), morning rest (MR), 2nd morning work (MW_2_), 1st afternoon work (AW_1_), afternoon rest (AR), and 2nd afternoon work (AW_2_) in each day’s wear-trial test.

### 2.4. Data Analysis

Descriptive statistical analysis on the WBGT and HR were conducted. The hourly WBGT and HR data were computed by averaging values measured each minute and every 5 s, respectively. The United States Military’s guidelines on WBGT suggest modifying activity in extreme heat conditions [34], and the Occupational Safety and Health Administration (OHSA) have used these guidelines to implement work-rest patterns for workers involved in manual labor jobs (e.g., construction workers, firefighters). The threshold temperatures are based on WBGT as follows: below 29.3 °C is low and low risk; between 29.4 °C and 32.1 °C is moderate risk; and WBGTs of above 32.1 °C is high risk. Maximal heart rate (HR_max_) is widely used as a criterion for achieving peak exertion in the determination of maximal aerobic capacity [35]. A percentage of the HR_max_ (%HR_max_) can be used to describe exercise intensity. The classification criterion for the HR used in this study was proposed by the ACSM [36]. Such classification is based on %HR_max_ as follows: below 54% is light; between 55% and 69% is moderate; and above 70% is high. Further analysis of hourly WBGT and %HR_max_ was also conducted based on the ACSM classification.

To establish the model for predicting CLP in hot weather, we used 15 min average direct work time, physiological parameters, and environmental parameters as model variables. 378 data sets of direct work time, environmental data, and physiological data were captured in total. 340 sets of data were employed to establish the CLP-thermal environment model, and the remaining 38 sets of data were adopted to validate the developed model. Multiple linear regression (MLP) was adopted to construct the association between one dependent variable (i.e., direct work time) and several independent variables (i.e., age, work duration, WBGT, cigarette intake, alcohol drinking consumption, weight, and work intensity). %HR_max_ was adopted to reflect the presence of work intensity. Because the steel-bar fixers were permitted to take water when they needed, and have been subjected to the hot environment for a long period, their hydration level and heat acclimatization level were not taken into account in the proposed model. Cigarette intake (“no”, “sometimes”, “usually”) and alcohol drinking consumption (“no”, “sometimes”, “usually”) were regarded as dummy variables for quantitative analysis in the regression model. The main purpose of “dummy variables” is to allow us to represent nominal-level independent variables in statistical techniques like regression analysis [37]. A stepwise selection process was adopted to build up the MLR model, since it carried out an automatic procedure to identify the best subset model [38]. Nevertheless, physical devices and instruments used for measurement are inevitably subject to transient failures, which can have a significant negative impact on the reliability and accuracy of the model. Thus, outliers more than 2 standard deviations from the average of each condition in the group were deleted by the SPSS block diagram program. A total of 32 outliers were identified and removed after the analysis. Multicollinearity is a phenomenon in which moderate or high inter-associations exist among the independent variables. Multicollinearity was detected by calculating the variance inflation factors (VIF), which quantifies the degree of multicollinearity among the predictors in regression analysis [39]. According to a rule of thumb [40], if the VIF is less than 10 there is no multicollinearity among factors. Standardization of the coefficient removes the original unit of measurement for variables in a regression equation, which enables us to compare the effect sizes of variables measured on different scales [41]. In order to test the validity of the model, 38 test samples were reserved to compute the factor scores for substitution into the multiple regression equation. RMSE, MAPE, and Theil’s inequality coefficient U were used to quantitatively measure the accuracy of the actual value of as well as the predicted value.

## 3. Results

### 3.1. Climatic Heat Stress

Table 2 shows the hourly WBGT data monitored in morning and afternoon work sessions. It can be seen that the hourly WBGT increased from 8:00 to 12:00 (morning session) and decreased from 13:00 to 17:00 (afternoon session). The lowest WBGT was 22.28 °C, which was found between 8:00 and 9:00. The highest WBGT was 35.46 °C, which was found between 13:00 and 14:00. Generally, WBGT values during the time close to 13:00 were higher than those of other periods.

**Figure 3:**
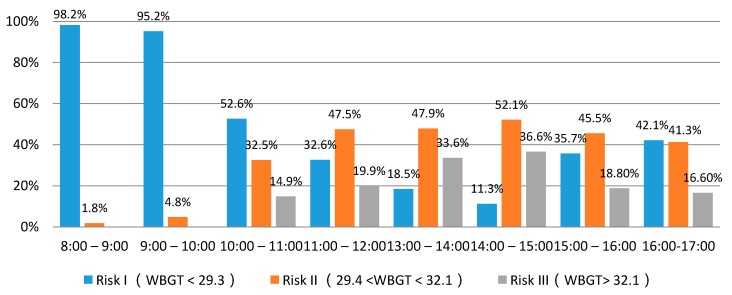
Distribution frequency of WBGT value in different time.

Further analysis on hourly WBGT distribution indicated that low risk, moderate risk and high risk account for 48.3%, 34.2%, and 17.5% of the day, respectively. Figure 3 shows the distribution frequency of WBGT values at different times. It can be seen that the frequency for both high risk and dangerous levels rose in the morning work session, approached the peak level between 14:00 and 15:00, and then declined in the afternoon work session. During the period from 8:00 to 10:00, more than 95% of the WBGT value was less than 29.3 °C. The highest occurrence frequency for both high risk and dangerous levels was observed from 13:00 and 15:00.

### 3.2. Heart Rate

Table 3 shows the hourly HR values measured at different times. It can be seen that the hourly HR ranged from approximately 100 to 110 bpm during the work, and from 90 to 95 bpm during the rest. Generally, the HR values during times close to 12:00 noon (10:15–12:00 and 13:00–15:00) were higher than those during other periods (8:00–10:00 and 15:00–16:30).

**Figure 4:**
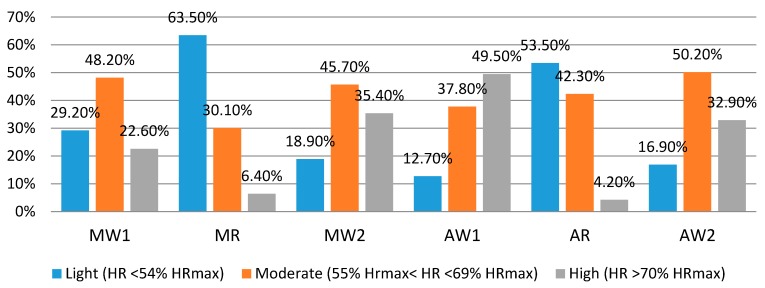
Distribution frequency of metabolic stress in different periods.

Further analysis on work intensity distribution indicated the low, moderate and high account for 24.0%, 43.7%, and 32.3% of the day, respectively. Figure 4 shows the distribution frequency of work intensity in different periods. During the rest periods (10:00–10:15 and 15:00–15:30), more than half of the %HR_max_ values are less than 54% (light work intensity). During the work periods, most %HR_max_ value belongs to 55–69% HR_max_ (moderate work intensity). It was found that the period of 13:00 and 15:00 has the highest frequency of high metabolic stress.

### 3.3. Construction Labor Productivity (CLP)

The CLP in terms of direct work time (DWT), indirect work time (IWT), and non-productive time (NPT) includes 4114 observations. Table 4 summarizes the descriptive statistics for DWT, IWT, and NPT in different periods. It can be seen that DWT takes up the most working periods (approximately 64%), followed by IWT (approximately 25%), and NPT (approximately 11%). During the morning work session, the proportion of DWT increases from 8:00 to 10:00, becomes stable between 10:00 and 11:00, and slightly reduces from 11:00 to 12:00 noon. During the afternoon work session, the proportion of DWT increases from 13:00 to 16:00. The highest DWTs emerged from 9:00 to 10:00 and 10:15 to 11:00. The lowest DWTs occurred from 14:00 to 15:00. The increase of DWT at the start of the work session might be explained by the fact that the workers need time to adapt to the tasks and work environments. The proportion of IWT decreases from the start in the morning, and reaches its lowest point after the 15-min break in the morning; then, it maintains its position in the afternoon until the end of the day. The relatively high percentage of IWT at the start of the work session may be workers needing time for material preparation. The trend of NPT is almost the opposite of DWT.

Table 5 shows the percentage of time spent on different tasks in DWT, IWT, and NPT. For direct work time, steel bar fixing tasks require approximately 91% of all DWT, whereas tasks concerned with the placement, adjustment, lifting, and measurement of steel bars are rare, accounting for nearly 9% of their time. For indirect work time, “walking for tools/materials” accounts for the largest proportion, with about 43.9% during all periods. “Discuss the work with foreman or each other”, “review the list of materials to understand the work”, “take materials” and “waiting for materials to be lifted” follow, taking up nearly 21.2%, 16.8%, 10.2%, and 5.3%, respectively. Because the workplace is limited in the field, due to the large number of rebars and stirrups, rebar workers have to carry steel bars to the jobsites many times throughout the day. For the NPT, the time spent on non-utilization is a little higher (52.5%) than the personal time spent on drink, chat, sit, go to toilet (47.5%). The non-utilization time is much higher than the personal time in the morning session, but much lower than the personal time in the afternoon work session.

### 3.4. Regression Model of CLP

The relationships between DWT and environmental, physiological, and personal factors were constructed by utilizing the stepwise selection regression technique. Table 6 shows the results of MLR analysis on DWT and six independent variables (i.e., age, WBGT, %HR_max_, alcohol drinking habits, work duration). The largest VIF of 5.68 was much lower than 10, indicating that multicollinearity did not increase the standard error of the CLP model estimates. The CLP-heat stress model with the adjusted R-squared of 0.68 was ultimately developed as Equation (2).

CLP = 1.602 − 0.028WBGT + 0.231·%HR_max_ − 0.035WD − 0.005Age − 0.085ADH (2)

where WBGT is wet bulb globe temperature (°C); WD is work duration (hours); ADH is alcohol drinking habit (“no consumption” = 0, one cup of red wine/white spirit/beer per day’ = 1, “two cups or more red wine/white spirit/beer per day” = 2), %HR_max_ is the percentage of maximum heart rate (%).

The model should be verified by using raw data, and the predicted results compared to the actual data to assess the predictive capacity of the model [42]. The RMSE, MAPE, and Theil’s U statistics were 0.857, 0.092, 0.009, which show that the developed CLP-heat stress model is highly predictable. Thus, the results of the assessment of the predicted values verify that the CLP-heat stress model is sufficiently effective and strongly predicts the CLP of steel bar fixers in Hong Kong.

## 4. Discussion

The unstandardized coefficient represents the amount by which a dependent variable will change if an independent variable is changed by one unit, while other independent variables remain constant. The regression results show that DWT increases with %HR_max_, but decreases with WBGT, work duration, age, and alcohol drinking habit. Differences in research results are usually derived from the different combinations of research components and the size of the population. Standardization of the coefficient is usually done to answer the question of which independent variables have a greater effect on the dependent variable in a multiple regression analysis, when the variables are measured in different units of measurement. According to the standardized coefficients, age, and alcohol drinking habits are the most important predictors to determine DWT.

The ageing workforce has long been recognized as a significant factor affecting productivity in many industrialized countries [43]. On the one hand, the ageing process involves many physical changes that make construction work tasks more difficult for older workers [44]. Some studies have shown that the elderly are more sensitive to the effects of temperature [6]. This may be due to the fact the thermal regulation system weakens with aging [45]. The elderly are less able to maintain homeostasis in response to environmental challenges [46]. On the other hand, older workers are more experienced and more familiar with the work, and hence more efficient. Li et al. revealed that working experience has positive influences on direct work time, but age has negative influences on direct work time [25]. The coefficient for “age” of CLP-heat stress model is negative, indicating that older workers are generally less productive than younger ones. Since most of the participants were young, aged between 21 and 39 years old, the factor of working experience plays a greater role in affecting labor productivity than that of age in this study.

Alcohol drinking habits influence work performance. Dzeng et al. evaluated the effect of alcohol on work performance [47]. It was found that alcohol significantly reduces the work quality and affects the ability to execute tasks [47]. The results of our study on the effects of alcohol consumption on labor productivity are consistent with this study. The coefficient for “alcohol drinking habit” is negative, which implies that drinking is bad for productivity and construction companies should discourage workers from drinking.

Our experiments found that as much as 17.5% of the work time in the hot summer months of Hong Kong are high risk, according to the US Army WBGT guidelines. This is a strong signal that in Hong Kong, as well as other regions with hot and humid weather, appropriate measures to reduce the potential risk of heat stress must be developed. The above analyses have presented a few causes for the high risk, which can help us identify countermeasures. In this study, we adopted the WBGT guidelines established by the U.S. Army to categorize the heat stress risk level. The WBGT guideline was used to modify basic training during extreme conditions. Although WBGT was recognized as the most widely used index to manage heat stress, and was revealed to have the highest validity in predicting the effects of heat stress on construction workers [27], there is no reference to national standards (e.g., ACGIH) or the International Standard for WBGT and the recommendations of construction work input. Thus, further WBGT guidelines for construction work should be developed by taking into account the intensity of construction work, site environment (e.g., in shade, outdoor, with or without cooling facilities), and the characteristics of construction workers.

HR is a physiological indicator of work intensity and heat stress [48]. Average HR was approximately 10 bpm lower for participants at rest (90–95 bpm) in comparison to those at work (100–110 bpm). The average HR during the time close to 12:00 noon was 10 bpm higher than that of other periods. The World Health Organization [49] indicated that people are at a high strain when his/her HR is greater than 160 bpm for brief periods. Minard et al. suggested that daily average heart rate should be less than 120 bpm [48]. The current findings suggested that the maximum HR of 161 bpm and average HR in the range of 90 and 110 bpm are within the proposed threshold. Average HR was approximately 10 bpm lower for participants at rest than for those at work.

It was found that the highest WBGT occurred between 13:00 and 14:00. To avoid exposure to a high level of heat stress, proper scheduling measures can be taken. For instance, construction managers could schedule physically demanding tasks to be carried out in the early morning, during which the WBGT is much lower [50]. Moreover, Hong Kong often rains during the summer. As rain falls from a higher and colder altitude, it is usually cooler than the ambient conditions at ground level. Additionally, wind chill can increase evaporation from its surface, cooling it quickly by removing the latent heat of evaporation. Therefore, it is also up to construction managers to keep track of the weather conditions on different days, and schedule strength-demanding jobs to be carried out on the cooler days. Further studies to propose an optimal construction schedule in different weather conditions are envisaged to be conducted.

The results show that the heart rate values during times close to 12:00 noon was higher than that of other periods. In other words, resting is helpful for workers to recover from heat stress, and recovery from heat stress could increase productivity. Then, a natural question is, what is the optimal timing and duration of rest for a work day in terms of maximizing productivity? Moreover, depending on the culture and the workers’ preferences, is it possible to extend the work day from 9 h (with a one-hour lunch break) to, e.g., 9.5 h, so that workers can have 30 min more rest? In addition, do the workers prefer starting work much earlier, say, at 6:00, and not working during the noon period (say, from 12:00 to 15:00)? For a large construction project, as there are many workers, it is possible to identify groups of workers with similar preferences and fulfill their preference. Again, how to optimally consider the workers’ preferences and design tailored schedules? All these questions are worthy of further research efforts.

## 5. Conclusions

Global warming has a profound effect on the construction industry, as construction workers are often exposed to heat stress, especially those working in tropical or subtropical areas. Excessive heat stress leads to health problems and lower productivity. Field studies were conducted to measure and monitor the environmental heat in terms of WBGT at construction sites during summer time. The results showed that in Hong Kong, due to its hot and humid weather, as much as 17.5% of the work time in the hot summer months is high risk. The Hong Kong Observatory issues Very Hot Weather Warnings to alert members of the public to the dangers of heat stroke and sunburn in very hot weather. The warnings released are based on the meteorological data collected from limited and scattered weather stations. The temperature on construction sites can reach higher than the outside air temperature, for example, for workers performing roof work, road construction or doing interior work on a building with no air conditioning and poor ventilation. Contractors are recommended to measure the onsite WBGT and issue an alert if the WBGT exceeds the threshold.

Taking rebar workers in Hong Kong as an example, we conducted a field experiment to examine the relationship between CLP and environmental heat (i.e., WBGT), work-related factors (i.e., work duration, work intensity), and workers’ personal information. The findings of our research indicate that age, alcohol drinking habits, percentage of maximum HR, work duration, and wet bulb globe temperature (WBGT), are good predictors in determining rebar workers’ labor productivity. The predictive adequacy of the CLP-heat stress model was further evaluated by RMSE, MAPE, and Theil’s U statistics. It demonstrated that the model is adequately robust. In light of the heat stress model, a set of good practices and indices can be developed to ensure the health and safety of site personnel working in hot weather. For example, since drinking alcohol lowers productivity, workers should not consume alcoholic and caffeinated drinks on a working day. More research should be carried out in the future, including into job scheduling considering temperature, work time design, and minimization of non-productive time.

## Figures and Tables

**Table 1:** Breakdown of direct, indirect, and non-productive work activities.

**A. Direct/Productive Work Activities**	
A-1	Make use of wrenches to connect, cut, band, and modify reinforcing steel bars
A-2	Place reinforcing steel bars
A-3	Modify reinforcing steel bars
A-4	Carry reinforcing steel bars
A-5	Use meter sticks for measurements
A-6	Bending
**B. Indirect Work Activities**	
B-1	Walk towards equipment, tools, materials
B-2	Wait for materials to be carried
B-3	Review the list of materials to understand the work
B-4	Talk with foreman and co-workers about the tasks
B-5	Take materials
**C. Non-Productive Activities**	
C-1	Employees or machines, or both, due to work stoppage from any cause
C-2	Chat, smoke, drink, sit, use cell phones, go to the washroom

**Table 2:** Descriptive statistics of the WBGT data in morning and afternoon work sessions.

Time	WBGT (°C)	
	Mean ± SD	Range
8:00–9:00	26.23 ± 1.23	22.28–28.36
9:00–10:00	27.48 ± 1.09	24.01–31.45
10:00–11:00	29.37 ± 1.98	26.73–34.22
11:00–12:00	30.23 ± 2.01	26.86–35.03
13:00–14:00	31.34 ± 2.02	26.07–34.59
14:00–15:00	31.87 ± 2.03	25.91–35.46
15:00–16:00	29.32 ± 1.99	25.32–34.64
16:00–17:00	29.24 ± 2.04	24.27–33.14

**Table 3:** Descriptive statistics of the HR data at different times.

Time	Status	HR (bpm)		%HR_max_ (%)	
		Mean ± SD	Range	Mean ± SD	Range
8:00–10:00	Work	101.63 ± 1.23	73–144	58.9 ± 7.8	36.7–72.7
10:00–10:15	Rest	92.92 ± 1.23	68–124	50.2 ± 5.5	34.3–62.3
10:15–12:00	Work	106.24 ± 1.23	71–158	64.2 ± 8.5	35.9–79.4
13:00–15:00	Work	109.37 ± 1.23	82–161	68.1 ± 9.3	41.6–80.5
15:00–15:30	Rest	94.31 ± 1.23	71–130	53.6 ± 6.6	36.7–66.3
15:00–16:30	Work	104.01 ± 1.23	83–156	66.2 ± 9.6	41.8–78.4

**Table 4:** Descriptive statistics of the CLP in DWT, IWT, and NPT.

Time	DWT (%)	IWT (%)	NPT (%)
	Mean ± SD	Mean ± SD	Mean ± SD
8:00–9:00	57.82 ± 10.34	28.46 ± 9.34	13.72 ± 4.12
9:00–10:00	70.53 ± 11.23	21.76 ± 8.29	10.71 ± 6.24
10:15–11:00	72.79 ± 8.49	17.87 ± 9.15	9.34 ± 6.73
11:00–12:00	67.41 ± 12.58	18.54 ± 10.22	14.05 ± 7.82
13:00–14:00	55.96 ± 13.07	29.05 ± 13.27	15.99 ± 9.23
14:00–15:00	59.35 ± 10.12	28.75 ± 12.16	12.90 ± 9.06
15:30–16:30	62.63 ± 11.21	27.31 ± 11.46	10.06 ± 8.04

**Table 5:** Percentage of time spent on different tasks in DWT, IWT, and NPT.

	8:00–9:00	9:00–10:00	10:15–11:00	11:00–12:00	13:00–14:00	14:00–15:00	15:30–16:30
A-1	91.20%	90.20%	89.30%	92.50%	88.90%	90.30%	91.40%
A-2	2.20%	2.50%	2.10%	2.40%	1.90%	2.60%	2.80%
A-3	1.00%	1.50%	1.90%	1.80%	1.20%	1.20%	1.70%
A-4	2.70%	1.90%	2.50%	2.00%	2.10%	3.20%	2.20%
A-5	2.90%	3.90%	4.20%	1.30%	5.90%	2.70%	1.90%
B-1	51.20%	46.80%	43.90%	50.20%	39.80%	41.10%	42.80%
B-2	6.80%	4.80%	3.80%	4.00%	4.30%	7.20%	4.30%
B-3	11.50%	10.60%	18.50%	13.80%	13.80%	19.60%	20.10%
B-4	20.70%	29.60%	23.60%	23.90%	29.90%	20.20%	23.40%
B-5	9.80%	8.20%	10.20%	8.10%	12.20%	11.90%	9.40%
C-1	67.30%	54.50%	76.20%	54.50%	34.20%	23.50%	22.50%
C-2	32.7%	45.5%	23.8%	45.5%	65.8%	76.5%	77.5%

**Table 6:** MLP analysis for CLP-heat stress model (*n* = 340).

	Unstandardized Coefficients	Standardized Coefficients (Rank)	Sig. *^p^*	Collinearity Statistics		Range
				Tolerance	VIF	
(Constant)	1.602		0.027			
WBGT	−0.028	−0.029	0.003	0.98	1.23	22.28–35.46
%HR_max_	0.231	0.058	0.021	0.63	1.65	34.3–80.5%
Work duration	−0.035	−0.099	0.009	0.45	2.12	0–4
Age	−0.005	−0.205	0.034	0.78	1.49	21–39
Alcohol drinking habit	−0.085	−0.143	0.045	0.36	3.25	0, 1, 2

*p* < 0.05.

## References

[1] Pachauri R.K., Reisinger A., IPCC, Core Writing Team (2007). Climate Change 2007: Synthesis Report. Contribution of Working Groups I, II and III to the Fourth Assessment Report of the Intergovernmental Panel on Climate Change.

[2] Hansen J., Sato M. (2016). Regional climate change and national responsibilities. Environ. Res. Lett..

[3] World Meteorological Organization The Global Climate 2011–2015: Heat Records and High Impact Weather. https://public.wmo.int/en/media/press-release/global-climate-2011-2015-hot-and-wild.

[4] Stocker T.F., Qin D., Plattner G.K., Tignor M., Allen S.K., Boschung J., Nauels A., Xia Y., Bex V., Midgley P.M., IPCC (2013). Climate Change 2013: The Physical Science Basis. Contribution of Working Group I to the Fifth Assessment Report of the Intergovernmental Panel on Climate Change.

[5] Yi W., Chan A.P.C. (2015). Effects of temperature on mortality in Hong Kong: A time series analysis. Int. J. Biometeorol..

[6] Vandentorren S., Bretin P., Zeghnoun A., Mandereau-Bruno L., Croisier A., Cochet C., Ribéron J., Siberan I., Declercq B., Ledrans M. (2006). August 2003 heat wave in France: Risk factors for death of elderly people living at home. Eur. J. Public Health.

[7] Hancher D.E., Abd-Elkhalek H.A. (1998). The effect of hot weather on construction labor productivity and costs. Cost Eng..

[8] Chan A.P.C., Yi W. (2016). Heat stress and its impacts on occupational health and performance. Indoor Built Environ..

[9] Occupational Safety and Health Administration 2013. OSHA News Release—Region 4. https://www.osha.gov/news/newsreleases/region4/05302013.

[10] National Safety Council Summer Heat Can Be Deadly for Construction Workers, CPWR Cautions. http://www.safetyandhealthmagazine.com/articles/15778-summer-heat-can-be-deadly-for-construction-workers.

[11] Chan A.P.C., Yam M.C.H., Chung J.W.Y., Yi W. (2012). Developing a heat stress model for construction workers. J. Facil. Manag..

[12] Construction Industry Council, Hong Kong Guidelines on Site Safety Measures for Working in Hot Weather. Publication Version 2. http://www.hkcic.org/eng/info/publication.aspx?langType=1033&id=4458.

[13] Department of Health, HKSAR Government Preventive Measures against Heat Stroke and Sun Burn. http://www.dh.gov.hk/english/press/2010/100706-3.html.

[14] Kjellstrom T., Briggs D., Freyberg C., Lemke B., Otto M., Hyatt O. (2016). Heat, human performance, and occupational health: A key issue for the assessment of global climate change impacts. Annu. Rev. Public Health.

[15] Kjellstrom T., Freyberg C., Lemke B., Otto M., Briggs D. (2017). Estimating population heat exposure and impacts on working people in conjunction with climate change. Int. J. Biometeorol..

[16] Dunne J.P., Stouffer R.J., John J.G. (2013). Reductions in labour capacity from heat stress under climate warming. Nat. Clim. Chang..

[17] Zander K.K., Botzen W.J.W., Oppermann E., Kjellstrom T., Garnett S.T. (2015). Heat stress causes substantial labour productivity loss in Australia. Nat. Clim. Chang..

[18] Altinsoy H., Yildirim H.A. (2015). Labor productivity losses over western Turkey in the twenty-first century as a result of alteration in WBGT. Int. J. Biometeorol..

[19] Grimm C.T., Wagner N.K. (1974). Weather effects on mason productivity. J. Constr. Div..

[20] Koehn E., Brown G. (1985). Climatic effects on construction. J. Constr. Eng. Manag..

[21] Thomas H.R., Yiakoumis I. (1987). Factor model of construction productivity. J. Constr. Eng. Manag..

[22] Auliciems A., Szokolay S.V. (1997). Thermal Comfort.

[23] Srinavin K., Mohamed S. (2003). Thermal environment and construction workers’ productivity: Some evidence from Thailand. Build. Environ..

[24] Li X., Chow K.H., Zhu Y., Lin Y. (2016). Evaluating the impacts of high-temperature outdoor working environments on construction labor productivity in China: A case study of rebar workers. Build. Environ..

[25] Jarkas A.M. (2010). The influence of buildability factors on rebar fixing labour productivity of beams. Constr. Manag. Econ..

[26] Gun R.T., Budd G.M. (1995). Effects of thermal, personal and behavioral factors on the physiological strain, thermal comfort and productivity of Australian shearers in hot weather. Ergonomics.

[27] Yi W., Chan A.P.C. (2015). Which Environmental Indicator Is Better Able to Predict the Effects of Heat Stress on Construction Workers?. J. Manag. Eng..

[28] Parsons K.C. (2003). Human Thermal Environments.

[29] Bernard T.E., Kenney W.L. (1994). Rationale for a personal monitor for heat strain. Am. Ind. Hyg. Assoc. J..

[30] Schlagbauer D., Heck D. (2013). Change in Output Performance due to Prolonged Work. ISEC 2013—7th International Structural Engineering and Construction Conference: New Developments in Structural Engineering and Construction 2013.

[31] Fox S.M., Haskell N.W.L. (1971). Physical activity and the prevention of coronary heart disease. Ann. Clin. Res..

[32] Maloney W.F. (1990). Framework for analysis of performance. J. Constr. Eng. Manag..

[33] Liou F., Borcherding J.D. (1986). Work sampling can predict unit rate productivity. J. Constr. Eng. Manag..

[34] Department of Army Heat Stress Control and Heat Causality Management. http://www.usariem.army.mil/assets/docs/partnering/tbmed507.pdf.

[35] Tanaka H., Monahan K.D., Seals D.R. (2001). Age-predicted maximal heart rate revisited. J. Am. Coll. Cardiol..

[36] ACSM Position Stand (1998). The recommended quantity and quality of exercise for developing and maintaining cardiorespiratory and muscular fitness, and flexibility in healthy adults. Med. Sci. Sports Exerc..

[37] Suits D.B. (1957). Use of Dummy Variables in Regression Equations. J. Am. Stat. Assoc..

[38] Wilcox R. (2012). More regression methods. Introduction to Robust Estimation and Hypothesis Testing.

[39] Mansfield E.R., Helms B.P. (1982). Detecting multicollinearity. Am. Stat..

[40] Kutner M. H., Nachtsheim C., Neter J. (2004). Applied Linear Regression Models.

[41] Freund R.J., Wilson W.J., Mohr D.L. (2010). Multiple Regression, Statistical Methods.

[42] Zang C., Schwingshackl C.W., Ewins D.J. (2008). Model validation for structural dynamic analysis: An approach to the sandia structural dynamics challenge. Comput. Methods Appl. Mech. Eng..

[43] Streb C., Voelpel S., Leibold M. (2009). Aging workforce management in the automobile industry: Defining the concept and its constituting elements. Gem. J. Res. Hum. Res. Manag..

[44] Fitzgerald M.D., Tanaka H., Tran Z.V., Seals D.R. (1997). Age-related declines in maximal aerobic capacity in regularly exercising vs. sedentary women: A meta-analysis. J. Appl. Physiol..

[45] Tian Z., Zhu N., Zheng G., Wei H. (2011). Experimental study on physiological and psychological effects of heat acclimatization in extreme hot environments. Build. Environ..

[46] Goggins W.B., Chan E.Y.Y., Yang C.Y. (2013). Weather, pollution, and acute myocardial infarction in Hong Kong and Taiwan. Int. J. Cardiol..

[47] Dzeng R.J., Wang S.H., Fang Y.C. (2015). Quantitative evaluation of the impact of night shifts and alcohol consumption on construction tiling quality. Appl. Ergon..

[48] Minard D., Goldsmith R., Farrier P.H., Lambiotte B.J. (1971). Physiological evaluation of industrial heat stress. Am. Ind. Hyg. Assoc. J..

[49] World Health Organization (WHO) (1969). Health Factors Involved in Working under Conditions of Heat Stress.

[50] Yi W., Chan A.P.C. (2015). Optimal work pattern for construction workers in hot weather: A case study in Hong Kong. J. Comput. Civil. Eng..

